# Bioactive and Nutritional Potential of Medicinal and Aromatic Plant (MAP) Seasoning Mixtures

**DOI:** 10.3390/molecules26061587

**Published:** 2021-03-13

**Authors:** Cláudia Novais, Carla Pereira, Adriana K. Molina, Ângela Liberal, Maria Inês Dias, Mikel Añibarro-Ortega, Maria José Alves, Ricardo C. Calhelha, Isabel C.F.R. Ferreira, Lillian Barros

**Affiliations:** Centro de Investigação de Montanha (CIMO), Instituto Politécnico de Bragança, Campus de Santa Apolónia, 5300-253 Bragança, Portugal; a32125@alunos.ipb.pt (C.N.); amolina@ipb.pt (A.K.M.); angela.liberal@ipb.pt (Â.L.); maria.ines@ipb.pt (M.I.D.); mikel@ipb.pt (M.A.-O.); maria.alves@ipb.pt (M.J.A.); calhelha@ipb.pt (R.C.C.); iferreira@ipb.pt (I.C.F.R.F.)

**Keywords:** condiment mixtures, phenolic compounds, organic acids, tocopherols, bioactivities, nutritional value

## Abstract

Medicinal and aromatic plants (MAPs), worldwide appreciated and used as condiments, dyes, and preservatives, possess several biological properties that justify their continuous application in the food industry. In the present study, the nutritional and chemical profiles, as well as the bioactive properties of four combinations of condiments, sold for seasoning poultry, meat, fish, and salads, were evaluated. Twenty-five phenolic compounds (HPLC-DAD-ESI/MS) were identified, with apigenin-*O*-malonyl-pentoside-hexoside as the major compound detected in all extracts. Oxalic and citric acids were identified in all mixtures (UFLC-PDA), as well as all the four tocopherol isoforms (HPLC-fluorescence). Regarding bioactivities, the mixtures for meat and salads (TBARS) and meat and poultry (OxHLIA) stood out for their antioxidant potential, whereas for the anti-inflammatory and antitumor properties, the mixtures revealing the greatest results were those for poultry and salad, respectively. In terms of antimicrobial activity, all the mixtures revealed the capacity to inhibit the growth of some bacterial strains. In brief, condiment mixtures showed to be a good source of bioactive compounds, as they confer health benefits, validating the importance of their inclusion in the human diet as a good dietary practice.

## 1. Introduction

The emergence of new diseases, degenerative conditions, and health problems related to physical inactivity, coupled with an increased life expectancy, have led to a growing intake of medicines. Nevertheless, the occurrence of several adverse effects related to the indiscriminate use of synthetic drugs has boosted the search for new and less aggressive treatments, often complementary to conventional ones [1]. Currently, herbal medicines are considered as low-cost preventive agents for various health issues, presenting low or practically non-existent toxicity, a high and proven effectiveness, the possibility of being taken orally, a well-known mechanism of action, and a good acceptance by the general and scientific community [2]. On the other hand, these plants capacity to prevent foodborne diseases and promote the extension of food shelf-life has led to their exploitation as sources of bioactive compounds to be employed in food industry, which mostly relies on artificial additives. 

Since ancient times, spices and MAPs have played an important role in human’s nutrition, imparting aroma, flavor, and color to food. Through their use as condiments, it is possible to reduce the use of salt and fat and, therefore, they are gaining increasing importance in good dietary practices [3]. Known as functional foods, these herbs not only meet common nutritional requirements, but also provide physiological benefits, being mostly composed of carbohydrates and a wide range of sugars, usually in low amounts, but also possessing a unique richness in other bioactive molecules [4,5]. 

Different groups of compounds are known to be present in MAPs, many of them having pharmacological properties, such as tocopherols and phenolic compounds, among others [4]. These latest have been considered one of the most important groups of natural and chemopreventive antioxidants, with potential anti-inflammatory, antidiabetic, and anticancer effects, and with cardio, hepatic, and neuroprotective actions, thus playing a crucial role in human health [6,7,8,9]. More importantly, these properties can be influenced by the action of other plant constituents, which can result in enhanced biological activities through synergistic or additive effects [10]. Together, these properties are of great interest in food, pharmaceutical, cosmetic, and other industries, promoting the health of the general population, both through their direct use in food and through their incorporation into different products, making them functional and biodynamic [11,12,13]. In this field, recent studies emphasize the efficiency of MAPs extracts in the development of bakery and dairy products, with functional properties for consumers’ health improvement [13,14,15]. At a more specific level, these plants can also be used as complementary therapies in the treatment of diabetes mellitus, given their recognized antidiabetic activity through different mechanisms of action [16], in inhibiting the proliferation of tumor cells, targeting enzymes and DNA transcription factors [17,18], and being important allies in the maintenance of human immune system, due to the presence of compounds with recognized anti-inflammatory potential [19].

Given these features, there is a global trend toward the development of herbal medicines from different mixtures of natural products, aiming at the standardization and a synergistic evaluation of the bioactive compounds present in these products [20,21,22]. On the other hand, given their widely appreciated taste, several mixtures have been prepared for seasoning purposes by a Portuguese company, using specific herbs that are more appreciated when applied to meat, poultry, salads, or fish. The plants selected for these mixtures’ preparation were *Allium schoenoprasum* L., *Petroselinum crispum* L., *Salvia officinalis* L., *Satureja montana* L., *Thymus vulgaris* L., *Thymus mastichina* L., *Rosmarinus officinalis* L., *Origanum vulgare* L., *Artemisia dracunculus* L., and *Thymus* × *citriodorus* L. Each mixture contains different proportion of 4 to 5 plants, according to their specific taste and traditional use. Thus, and given their wide use in Portuguese cuisine, the aim was to characterize these four mixtures of condiments both chemically and nutritionally and evaluate their bioactive properties, corroborating the importance of their inclusion in daily diet and in complementary therapeutic applications. 

## 2. Results and Discussion

MAPs are worldwide established as nutritious plants commonly present in the Mediterranean diet. In the present study, four mixtures of condiments were assessed for their nutritional, chemical, and bioactive properties. These mixtures were created and are commercialized by the Portuguese company Cantinho das Aromáticas. The plants were selected and combined according to their specific taste and traditional use, but their relative proportion in the mixtures is protected for commercial reasons. In terms of qualitative composition, the mixture for poultry consists of *Allium schoenoprasum* L., *Petroselinum crispum* L., *Salvia officinalis* L., *Satureja montana* L., and *Thymus vulgaris* L.; the mixture for meat is composed of *P.crispum*, *S. officinalis*, *Thymus mastichina* L., and *Rosmarinus officinalis* L.; the mixture for salads consists of *A. schoenoprasum*, *P. crispum*, *Origanum vulgare* L., and *T. mastichina*; and the mixture for fish is composed of *A. schoenoprasum*, *Artemisia dracunculus* L., *P. crispum*, and *Thymus* × *citriodorus* L.

### 2.1. Nutritional Value 

The results reached for macronutrients of the studied mixtures are shown in Table 1. Since all the analyzed condiments are mixtures of plants whose only qualitative composition is known and no information is provided regarding their proportion in each mixture, the comparison of the results obtained was made, whenever possible, considering the available literature for the individual plants. Carbohydrates were the major macronutrients found in all mixtures, with values ranging from 65.2 to 72.0 g/100 g dw for the mixtures for fish and salad, respectively. These macronutrients can function as signaling, recognition, and adhesion molecules, being involved in many important physiological functions, such as normal embryonic development, growth, host–pathogen interaction during infection, and development of diseases and metastases, among others [4]. Proteins are also present in considerable amounts (15.5 to 20.9 g/100 g dw), being an important nutrient that provides a basis of energy and essential amino acids for the growth and maintenance of the human health, once they may have specific biological activities that prevent some diseases, particularly at a cardiovascular and bone level, while promoting weight control and satiety [23].

### 2.2. Chemical Composition

#### 2.2.1. Free Sugars

Free sugars were also analyzed, and the results are presented in Table 2. Fructose, sucrose, and glucose were detected in all mixtures, with that for salad showing the highest total sugars (8.4 g/100 g dw) and the mixture for meat the slightest (4.28 g/100 g dw). In a previous study [4] where several condiments were analyzed, *A. schoenoprasum* was found to be the plant with the highest fructose content, which could explain the low amount detected in the mixture for meat, once this plant is not present in its constitution. Also, the same authors found that *T. citriodorus,* present in the seasoning mixture for fish, holds high amounts of sucrose, thus influencing its greater values. The small concentration of sugars is part of a minimal share of carbohydrates, making these condiments suitable for a low-calorie, balanced, and diversified diet.

#### 2.2.2. Organic Acids

The results achieved for organic acids are shown in Table 3, with the mixture for meat presenting the highest total concentration (4.17 mg/100 g dw), followed by those for poultry, fish, and salad (3.65, 3.6, and 2.63 mg/100 g dw, respectively). Malic acid was found to be the most abundant organic acid in all samples, with higher concentration in the mixture for meat (2.34 mg/100 g). Once P. crispum is present in all the studied mixtures, and given the fact that malic acid is also prevalent in this species, its higher concentration in the mixtures may be related to its presence [24]. Also, T. mastichina, present in the mixture for meat, presents malic acid in large amounts, which may also contribute to its greater evidence in this sample. On the other hand, citric acid showed the lowest values in the mixtures of poultry and meat (0.95 and 0.88 mg/100 g dw, respectively), not being detected in the mixture for salad. Also, fumaric acid was only detected in trace amounts in the mixtures for poultry and meat. Thus, in addition to the ability of preventing oxidative stress, these extracts could also be included in food formulations as acidulants, given the presence of citric and malic acids in their composition [25]. These compounds have also been reported to be responsible for cardioprotective effects, in which adjacent mechanisms may be related to their anti-inflammatory and antiplatelet effects [26]. Other authors reported that malic acid, the major organic acid present in all of the studied mixtures, has good antioxidant properties and is able to reduce cell apoptosis [27]. It also demonstrated antimicrobial activity against Listeria monocytogenes, Salmonella Enteritidis, and Escherichia coli O157:H7. On the other hand, fumaric acid derivatives have shown effectiveness against psoriasis and in the prevention of cardiovascular and diabetic diseases, as well as citric acid, which can also improve ketosis and protect against the development of diabetic complications [28]. In addition, these compounds act as precursors of phenolic and flavoring compounds [29].

#### 2.2.3. Tocopherols

Regarding tocopherols, the analysis allowed the identification of the four isoforms (α-tocopherol, β-tocopherol, γ-tocopherol, and δ-tocopherol) in all of the analyzed extracts (Table 4), with the mixtures for fish, meat, and salad presenting the highest total tocopherol values (17.1, 17.0, and 16.6 mg/100 g dw, respectively), and that for poultry showing a lowest amount (14.5 mg/100 g dw). 

γ-tocopherol was the most abundant isoform found in all the samples in the study, with higher concentrations in the mixtures for fish and salad (14.1 and 13.85 mg/100 g dw, respectively) and the lowest in the mixture for poultry (8.17 mg/100 g dw). In its turn, δ-tocopherol was the least predominant isoform, with concentration values ranging from 0.80 mg/100 g dw for the mixture for poultry to 0.134 mg/100 g dw for the mixture for seasoning meat. The mixture for salad is composed of O. vulgare and T. mastichina, characteristic for their composition in tocopherols, namely α-tocopherol and γ-tocopherol, in considerable amounts [30], which may explain the high incidence of γ-tocopherol in this mixture. However, the same is not observed for α-tocopherol, which may be due to the different concentrations of these and other plants used in the mixture composition. The mixture for meat, which also has T. mastichina and R. officinalis in its composition, rich in γ-tocopherol, as previously mentioned [31], may be responsible for the values presented, together with other species present in the mixture. In addition to the radical scavenging action of tocopherols, non-antioxidant functions have also been shown by these compounds, both in vivo and in vitro [32,33].

#### 2.2.4. Fatty Acids

The lipid fraction in MAPs is generally low and data on the composition of fatty acids is essential, especially with respect to the content of polyunsaturated fatty acids (PUFA) and saturated fatty acids (SFA) [34]. The fatty acid composition of the mixtures under study is shown in Table 5. 

To the best of our knowledge, there is no available data allowing the comparison of the results obtained herein with those reported by other authors. A total of 23 different fatty acids were identified, which reflect a good fatty acids profile and corroborates the importance of the inclusion of these condiments in a balanced diet. Additionally, it is worth highlighting the prevalence of PUFA (between 57 and 65.8%) compared to SFA (between 30.5 and 39%). In all mixtures, the SFAs found in greater quantity were palmitic (C16:0) and stearic acid (C18:0), whereas the mostly present MUFA (monounsaturated fatty acids) were palmitoleic (C16:1) and oleic (C18:1n9c) acids. Finally, the most prevalent PUFAs were linoleic acid (C18:2n6c), in values ranging from 18.6 to 21.47%, and α-linolenic acid (C18:3n3; 36 to 42.7%). Given the important role of linoleic and α-linolenic acids as precursors of omega-6 and omega-3 fatty acids, and the fact that these compounds cannot be synthesized in the human organism and must be obtained from the diet [4], it is of great interest to verify its presence in the studied mixtures.

#### 2.2.5. Phenolic Compounds

The tentative identification of the phenolic compounds found in the four condiment mixtures, as well as the retention times (Rt), maximum absorbance (λ_max_), pseudomolecular ion ([M-H]^−^), and the main ion fragments (MS^2^) of each phenolic compound are presented in Table 6. The attempt to identify the individual phenolic compounds was based on the data presented and, whenever possible, in comparison to the available standard compounds and/or with the existing literature. Among the twenty-five compounds detected and tentatively identified, thirteen were phenolic acids and eleven were flavonoids. Peaks **5**, **10**, **14**, **15**, and **16** were tentatively identified by comparing their retention time and UV spectrum with available standards, as caffeic acid, quercetin-3-*O*-glucoside, luteolin-7-*O*-glucoside, and cis and trans rosmarinic acid, respectively. Particularly, for peaks **15** and **16**, the standard compound presented the same retention time of peak **16**, identified as the trans isoform, and peak **15** (with the same chromatographic response as the previous peak) as the cis isoform. Regarding phenolic acids, peaks **2** ([M-H]^−^ at *m/z* 353), **3** ([M-H]^−^ at *m/z* 341), **6/7** ([M-H]^−^ at *m/z* 325), **11** ([M-H]^−^ at *m/z* 421), **12** ([M-H]^−^ at *m/z* 521), **17** ([M-H]^−^ at *m/z* 717), **19** ([M-H]^−^ at *m/z* 537), **23** ([M-H]^−^ at *m/z* 493) and **24/25** ([M-H]^−^ at *m/z* 557) were tentatively identified by comparing their chromatographic responses with the previously described [35,36], being therefore tentatively identified as 3-*O*-caffeoylquinic acid, caffeic acid hexoside [37], p-coumaric acid hexoside [37], 4-Hydroxy-7-*O*-(3-hydroxy-4-*O*-glucosylbenzoyl)benzyl alcohol [38], rosmarinic acid hexoside [39], salvianolic acid B [39], lithospermic acid A, salvianolic acid A [39], and *p*-coumaric acid isomers I/II [40], respectively. The group of the flavonoid compounds, peaks **4** ([M-H]^−^ at *m/z* 593), **9** ([M-H]^−^ at *m/z* 1093), and **13** ([M-H]^−^ at *m/z* 461) were also tentatively identified by comparing their chromatographic response with the previously described in the literature, being therefore identified as apigenin-*C*-dihexoside [41], kaempferol-*O*-glycosyl-(*p*-coumaroyl-hexosyl)-hexosyl-glucuronide [42], and luteolin-7-*O*-glucuronide [37]. The remaining peaks belonging to the group of flavonoids were tentatively identified by analyzing their pseudomolecular ion and MS^2^ fragments. Peak **8** presents a pseudomolecular ion [M-H]^−^ at *m/z* 725 and fragments MS^2^ at *m/z* 681 (44 u), 519 (162 u), 357 (162 u), 315 (42 u, isorhamnetin aglycone), which corresponds to the loss of a malonyl group and two hexosides, being tentatively identified as isorhamnetin-*O*-malonyl-dihexoside. Peak **18** ([M-H]^−^ at *m/z* 593) showed a single MS^2^ fragment at *m/z* 285 (luteolin aglycone, 308 u), and was tentatively identified as luteolin-7-*O*-rutinoside. Peaks **20** and **21**, present a very similar chromatographic response, with a pseudomolecular ion [M-H]^−^ at *m/z* 649 and with MS^2^ fragments at *m/z* 605 and 563 (44 + 42 u, malonyl group), and 269 (132 + 162 u, pentosyl and hexosyl groups) being therefore tentatively identified as apigenin-*O*-malonyl-pentosyl-hexoside. Peak **22**, shows a pseudomolecular ion [M-H]^−^ at *m/z* 679 with MS^2^ fragments at *m/z* 635 and 593 (44 + 42 u, malonyl group) and 299 (132 + 162 u, pentosyl and hexosyl groups), being tentatively identified as diosmetin-*O*-malonyl-hexosyl-pentoside. Finally, one flavan-3-ol was identified (peak **1**), with a pseudomolecular ion [M-H]^−^ at *m/z* 305, a maximum absorption spectrum at 270 nm and MS^2^ fragments characteristic of a (epi) galocatechin, being tentatively identified based on the previously described by Bouziane et al. [43].

The quantification data of the phenolic compounds present in the analyzed extracts are shown in Table 7. The mixture for meat showed the highest concentration in phenolic compounds (101.2 mg/g), followed by those for fish (76.1 mg/g) and for poultry (68.2 mg/g), with the extract of the mixture for salad presenting the lowest content in phenolic compounds (52.1 mg/g). In all extracts, the most abundant compound is apigenin-O-malonyl-pentosyl-hexoside, which may be due, once again, to the presence of P. crispum in all of the mixtures, since it is a predominant aglycone in this plant, as well as luteolin, quercetin and isorhamnetin, also present in considerable amounts [44,45]. Regarding phenolic acids, the most prevalent was rosmarinic acid (cis and trans isoforms), being more abundant in mixtures for meat and poultry. 

### 2.3. Bioactive Properties

#### 2.3.1. Antioxidant Activity

The antioxidant activity of the hydroethanolic extracts of the seasoning mixtures was assessed through the determination of their ability to inhibit lipid peroxidation and oxidative hemolysis. For that purpose, two in vitro assays were performed: TBARS and OxHLIA, respectively. The results obtained were presented as IC_50_ values (μg/mL), which correspond to the extract concentration needed to achieve 50% inhibition of lipid peroxidation and oxidative hemolysis, respectively. The lower the IC_50_ value, the greater the antioxidant and antihemolytic activity of the extracts. The results are shown in Table 8, where significant differences between the evaluated extracts are expressed. The extracts from the mixtures for meat and salad presented higher antioxidant activity, with IC_50_ values of 4.8 µg/mL and 6.6 µg/mL, respectively. The remaining extracts also demonstrated the ability to inhibit lipid peroxidation, but higher concentrations were required to obtain the same effect, with a maximum of 27 µg/mL for the mixture for poultry. Also, in the OxHLIA assay, the mixture for meat extract demonstrated the ability to protect 50% of the erythrocyte population from oxidation caused by AAPH (2,2′-azobis(2-methylpropionamidine) dihydrochloride; the oxidizing agent), although for only 60 min, with an IC_50_ value of 13 µg/mL, showing no activity at 120 min. It is also important to highlight that the mixture for poultry revealed activity at lower concentrations than Trolox (21.8 µg/mL), the positive control, with an IC_50_ value of 16.5 µg/mL, similar to that of the mixture for meat. The same antioxidant capacity was verified for the other extracts, although in higher concentrations, with a delay of 60 min in hemolysis. However, in this case, the mixture for fish was the one that needed the highest concentration to express some activity (IC_50_ of 106 µg/mL). The mixture for salad extract showed an IC_50_ value higher than that of Trolox, but it was also the only extract revealing the capacity to delay the oxidative hemolysis for 120 min.

In general, all extracts revealed a great antioxidant capacity, which, according to several authors, in plants, is mainly associated with the molecules like phenolic compounds, organic acids, and tocopherols [7,29,46]. Other authors also correlate the antioxidant activity found in plant extracts with apigenin, in this case present as apigenin-*O*-malonyl-pentoside-hexoside in all the extracts, in high concentrations. Apigenin is an aglycone that holds several biological assets, including antioxidant, anti-inflammatory, antitumor, antigenotoxic, antiallergic, neuroprotective, cardioprotective, and antimicrobial activities [44]. Notable biological effects are also attributed to rosmarinic acid, namely antioxidant properties [47], which is mostly present in the mixtures for poultry and meat that exhibited the greatest capacity to inhibit the oxidative hemolysis, in OxHLIA assay. Significant differences were observed in the antioxidant activity between the performed assays, mainly regarding the mixture for poultry, which, in the TBARS assay, revealed the worst ability to inhibit lipid peroxidation compared to the other extracts. Though, for OxHLIA assay, this extract proved to be one of the most effective ones, with an IC_50_ value lower than that of Trolox. These discrepancies may be due to the concentration of tocopherols in this mixture, which, according to our results, was lower than in the other mixtures. Tocopherols are known to have an important role in preventing lipid peroxidation, herein demonstrated by the inhibition of formation of TBARS, a direct consequence of this capacity [48]. Despite the highest IC_50_ values found for the extract, comparing with Trolox, it is important to note that pure compounds generally reveal more activity than extracts, especially this specific water-soluble vitamin E derivative, which holds an extraordinary antioxidant activity. Thus, the IC_50_ values obtained with the extracts, where each of the antioxidant compounds is present in a lower final concentration, are great results.

#### 2.3.2. Antimicrobial Activity

In this study, antibacterial analysis was performed by means of eight bacterial strains of clinical interest and the results are shown in Table 9. In general, all extracts showed activity against the studied bacteria, except for *Klebsiella pneumoniae* and *Pseudomonas aeruginosa*, for which a concentration higher than 20 mg/mL would be required to exert inhibitory activity, for all the extracts. The extracts revealing the most promising results were those of the mixtures for meat and for salad, which revealed MIC values of 2.5 mg/mL for *Enterococcus faecalis*. This bacterium, together with *Escherichia coli*, revealed the highest sensitivity for the mixture of fish extract (MIC value of 5b mg/mL). For all the assessed bacteria, the minimum bactericidal concentration (MBC), after further evaluation of microbial growth, was found to be higher than 20 mg/mL for all the extracts. This observation could indicate that the antimicrobial compounds present in the extracts mainly hold inhibitory activity, with higher concentrations required for a possible bactericidal activity. It should be noted that, in this work, microorganisms were obtained from clinical isolates, which often have greater resistance to antibiotics, compared to commercial ones. 

MAPs are known to be wealthy in several compounds with antimicrobial properties, with organic acids playing an important role in this field by preserving the quality and organoleptic characteristics of fruits and vegetables [49]. In general, all extracts were effective against the bacteria under study, and no relationship between the more complex constitution of the cell wall of Gram-negative bacteria and their effectiveness was evidenced. The extracts from the mixtures for meat and salad showed, in general, the best antimicrobial activity for all strains and, considering the possible antagonistic and/or synergistic effects of the different bioactive compounds present in these mixtures, malic and oxalic acids may be the main responsible for the activity of these extracts. Previous studies have identified different organic acids from Japanese apricot fruits and determined antimicrobial activities against *E. coli*, *Bacillus subtilis,* and *Staphylococcus aureus* [50]. Other authors reported the antimicrobial activity of oxalic acid against nine different phytopathogenic bacteria, with a bactericidal effect [51]. In addition, phenolic compounds may also contribute to the antimicrobial activity presented by the extracts [9], where quercetin and isorhamnetin, present in greater quantities in the extracts with greater antimicrobial potential, may be those that mostly influence this bioactivity. These compounds are recognized not only for their antimicrobial activity, but also by their antioxidant, anti-inflammatory, and anticancer properties [52,53]. Although many studies seek to isolate the active components of these plants, the effect of an herbal medicine is usually explained by the synergistic action between the compounds. This interaction can occur in such a way as to enhance its antimicrobial action, or it can promote the reduction and even the loss of this activity [10].

#### 2.3.3. Anti-Inflammatory Activity

The IC_50_ values reached by assessing the ability of the hydroethanolic extracts of the mixtures to inhibit 50% of nitric oxide (NO) production in the mouse macrophage cell line (RAW 264.7) are presented in Table 10. Generally, all the extracts showed lower efficiency than the positive control (dexamethasone), demonstrating, however, anti-inflammatory potential, except for the extract of mixture for salad, which did not show activity at the maximum tested concentration (400 µg/mL). The mixtures for poultry and fish showed the most effective results, with a concentration of 54 µL/mL and 59 µg/mL, respectively, required to promote 50% inhibition of NO production. The mixture for salad, on the other hand, did not reveal activity at the highest tested concentration (400 µg/mL).

Several authors reported that apigenin derivates have the capacity to inhibit organism’s inflammatory response [44,54], which indicates that apigenin-*O*-malonyl-pentoside-hexoside, present in high concentrations in all the studied extracts, has possibly enhanced the anti-inflammatory effect of the extracts. Additionally, luteolin, which derivatives were found in the studied extracts as luteolin-7-*O*-glucuronide, luteolin-7-*O*-glucoside, and luteolin7-*O*-rutinoside, is widely known for being responsible for several pharmacological activities. Its anti-inflammatory activity is partially related to the regulation of inflammatory mediators and various cytokines in in vitro and in vivo models [55,56]. Plants with larger amounts of luteolin have long been used in Irani, Brazil, and in traditional Chinese medicines to treat diseases related to inflammation [57,58]. Different types of luteolin-mediated regulation of inflammatory mediators have already been described, namely in RAW 264.7 macrophages [59,60,61]. The lower anti-inflammatory activity observed in the mixture for meat, in which a higher content of phenolic compounds was identified, may be due to antagonistic effects between the various compounds present in the extract, although further studies would be needed to prove this hypothesis. It is worthy to remember that the countless biological activities ascribed to MAPs mostly result from the action of their diverse constituents or synergisms between them, constituting a rich ethnopharmacological heritage [10].

#### 2.3.4. Antitumor Activity

The extracts obtained from the different mixtures were also tested for their antitumor activity against five tumor cell lines (Table 10). The results are expressed as GI_50_ values, translating the extract concentration that provides 50% of cell growth inhibition. All extracts showed a GI_50_ value higher than that of the positive control (elipticin). However, it was observed that, except for the mixture for meat (for the NCI-H460 line), all extracts showed effective results in inhibiting the growth of the tested cell lines. The mixture for salad showed the lowest GI_50_ values for all the cell lines, particularly for MCF-7 (10.54 µg/mL). In general, MCF-7 cell line was the most sensitive to all the tested extracts, in contrast with NCI-H460 cell line that required higher extract concentrations. Overall, the mixture for salad showed to be the most effective in inhibiting tumor growth in all the tested cell lines.

Several epidemiological studies have been consistently reporting an inverse association between the intake of phenolic compounds, particularly those derived from phenolic acids and flavonols, and the risk of cancer in humans. Subsequently, reviews on cancer prevention and treatment in animal and cell line models have been reflecting the effectiveness of phenolic compounds in inhibiting cancer development. Additionally, these compounds are described as having different mechanisms of action against tumor cells, namely through targeting human cell receptors, enzymes, transcription factors, and others [18]. Quercetin derivatives, widely occurring in plants and present in the studied extracts have been attracting further attention due to their anticancer activity, as well as antioxidant, antiviral, antibacterial, and anti-inflammatory effects [52]. These compounds are also known to inhibit the proliferation of various cell lines related to human breast cancer due to its pro-oxidant capacity, which contributes to the prevention of tumor growth and stimulates the induction of apoptosis, interrupting the normal cell cycle [62]. The presence of this phenolic compound in the studied extracts supports the results obtained in MCF-7 cell line, which revealed a high sensitivity to all the extracts. The luteolin derivates detected in the extracts may also be responsible for their pharmacological activities. According to previous studies, these compounds exert anticancer effects, inducing apoptosis, interrupting the cell cycle, and inhibiting metastasis and angiogenesis in several cancer cell lines, such as breast, colon, pancreas, and lung cell lines, among others [63,64,65]. The effects of apigenin in cancer prevention may be due to its potent antioxidant and anti-inflammatory activities, investigated in studies carried out in ovarian and breast cancer and in the risk of recurrence of neoplasia in colorectal cancer [44,54,66,67]. The great antitumor activity presented by the mixture for salad extract is not, however, exclusively linked to the compounds derived from quercetin and luteolin, since they are present in similar concentrations in all the studied extracts. Furthermore, this mixture has the lowest total content of phenolic compounds, which can lead to conclude that its activity is also ascribed to the remaining bioactive compounds assessed (e.g., organic acids and tocopherols). The action of these compounds, either individually or in interaction with each other, generating antagonistic, synergistic, or additive effects, make it difficult to predict their effects in trials with different cell lines [10]. Also, flavonoids can be an important complement in the prevention and treatment of several types of cancer, due to their natural origin, safety, and low cost in relation to synthetic drugs. However, as most of the findings cited in the present work are based on in vitro and in vivo studies, they do not necessarily represent this effect in humans. Thus, further investigations would be necessary to complement this study, focusing the mechanisms of action of these compounds in cancer prevention/treatment.

## 3. Materials and Methods

### 3.1. Samples

The samples of seasoning mixtures were provided by Cantinho das Aromáticas (Vila Nova de Gaia, Porto), in the dry state. Their storage was carried out in a dry place and protected from light. The mixture for poultry consists of *Allium schoenoprasum* L.*, Petroselinum crispum* L.*, Salvia officinalis* L.*, Satureja montana* L., and *Thymus vulgaris* L.; the mixture for meat is composed of *P.crispum*, *S. officinalis*, *Thymus mastichina* L., and *Rosmarinus officinalis* L.; the mixture for salads consists of *A. schoenoprasum*, *P. crispum*, *Origanum vulgare* L., and *T. mastichina*, and the mixture for fish is composed of *A. schoenoprasum*, *Artemisia dracunculus* L., *P. crispum*, and *Thymus* × *citriodorus* L.

### 3.2. Extract Preparation

Hydroethanolic extractions were performed by maceration of the dry material for the analysis of the phenolic composition and bioactive properties, as previously described by Barros et al. [68]. Briefly, 1.5 g of sample was subjected to an extraction of 1 h (25 °C at 150 rpm) twice with 40 mL of ethanol (Sigma-Aldrich, St. Louis, MO, USA)/water (80:20; *v/v*) and, then, filtered through Whatman No. 4 paper. The ethanol of the combined extracts was removed using a rotary evaporator (Büchi R-210, Flawil, Switzerland) and the extract was frozen and lyophilized (FreeZone 4.5 model 7750031, Labconco, Kansas City, MO, USA) for further analysis. 

### 3.3. Chemical Composition

#### 3.3.1. Proximate Composition and Energetic Value

According to the Official Methods of Analysis of AOAC [69], the proximate composition was determined and expressed in g/100 g dry sample. The incineration at 550 ± 5 °C was used to determine the ash content. Crude protein was estimated by the macro-Kjeldahl method (N × 6.25) using an automatic distillation and titration unit (model Pro-Nitro-A, JP Selecta, Barcelona). Soxhlet extraction was used to determine the crude fat, with petroleum ether during 7 h. Total carbohydrates content was calculated by difference: Total carbohydrates (g/100 g) = 100 − (fat + g ash + g proteins). The energetic value was calculated according to the Atwater system using the formula: Energy (kcal/100 g) = 4 × (g proteins + g carbohydrates) + 9 × (g fat).

#### 3.3.2. Sugars

The extraction of free sugars from the dry samples was carried out according to Barros et al. [68] The compounds were identified by high performance liquid chromatography with a refraction index detector (HPLC-RI; Knauer, Smartline 1000 and Smartline 2300 systems, respectively) operating as previously described by the authors. Peaks identification was performed by comparisons of their relative retention time (Rt) with authentic standards. Quantification was completed using melezitose as IS, (Sigma-Aldrich, St. Louis, MO, USA). Results were processed in a Clarity Software (Data Apex, Prague, Czech Republic) and expressed in g per 100 g dw. 

#### 3.3.3. Organic Acids

The analysis of organic acids followed the protocol established by the group [70]. The evaluation was performed by ultra-fast liquid chromatography coupled to a photodiode array detector (UFLC-PDA; Shimadzu Coperation, Kyoto, Japan). The separation of the compounds was carried out in a C18 SphereClone (Phenomenex) reverse phase column (5 μm, 250 × 4.6 mm id) thermostated at 35 °C, using 3.6 mM sulfuric acid solution as an eluent at a flow rate of 0.8 mL/min. The identification was carried out by comparing the chromatograms obtained for the analyzed samples with those obtained using commercial standards. The quantification of the compounds was done by relating the peak areas, recorded at 215 nm, with the calibration curves obtained with commercial standards for each compound. The results are presented in mg per 100 g dw.

#### 3.3.4. Tocopherols

For the determination of tocopherols, the methodology was applied according to that previously described by the authors [68]. The previously described HPLC system was used, coupled to a fluorescence detector (FP-2020; Jasco, Japan) programmed for excitation at 290 nm and emission at 330 nm. The separation of the tocopherol isoforms was achieved using a normal phase column of Polyamide II (250 mm × 4.6 mmi.d.) from YMC Waters (Japan), operating at 30 °C. The mobile phase used was a mixture of hexane and ethyl acetate (7: 3, *v/v*) with a flow rate of 1 mL/min and an injection volume of 20 µL. Quantification was based on the response of the fluorescence signal, using the internal standard method and by chromatographic comparison with standards. Tocol (Matreya, Pleasant Gap, State College, PA, USA) was used as internal standard, and the results were expressed in mg/100 g dw.

#### 3.3.5. Fatty Acids

The fatty acids were determined after the trans-esterification process, as previously described by Barros et al. [68]. The analysis was made using a gas chromatographer DANI model GC 1000 instrument equipped with a split/splitless injector and a flame ionization detector (GC-FID, 260 °C). The identification and quantification of the compounds were performed by comparing the relative retention times of fatty acid methyl ester (FAME) from commercial standards. Fatty acids were processed using Clarity Software (DataApex 4.0, Prague, Czech Republic) and the results expressed in relative percentage.

#### 3.3.6. Phenolics Compounds

The phenolic compounds were evaluated in the lyophilized hydroethanolic extracts and redissolved in ethanol/water (80:20; *v/v*) to a final concentration of 10 mg/mL. The evaluation was performed using a Dionex Ultimate 3000 UPLC (Thermo Scientific, San Jose, CA, USA), equipped with a DAD detector (280 and 370 nm as the preferred wavelength) and coupled to an electrospray ionization mass detector (LC-DAD-ESI/MSn). The chromatographic separation of the compounds was performed with a Waters Spherisorb S3 ODS-2 C18 column (3 µm, 4.6 mm × 150 mm, Waters, Milford, MA, USA), operating at 35 °C. The elution solvents, working in the gradient, were 0.1% formic acid in water and acetonitrile. Finally, for detecting MS in negative mode, a Linear Ion Trap LTQ XL mass spectrometer (ThermoFinnigan, San Jose, CA, USA) equipped with an electrospray ionization source (ESI) was used. The identification of phenolic compounds was performed based on chromatographic behavior, spectra, and UV-Vis masses, by comparison with standard compounds or data previously described in the literature, using the Xcalibur^®^ software (ThermoFinnigan, San Jose, CA, USA). Quantitative analysis of the identified compounds was performed using calibration curves based on the UV signal of the standard compounds. When commercial standards were not available, the calibration curves of the most similar standards were used. The operating conditions were previously described in detail by Bessada et al. [71] as well as the identification and quantification procedures. The results are expressed in mg/g extract.

### 3.4. Bioactive Properties

#### 3.4.1. Antioxidant Activity

The antioxidant activity of the extracts obtained from the four mixtures was evaluated by means of two cell assays: oxidative hemolysis inhibition assays (OxHLIA) and the lipid peroxidation inhibition by thiobarbituric acid reactive substances assay (TBARS), according to the procedure reported in detail by Lockowandt et al. [72]. The results were expressed in IC_50_ values, meaning the concentration of extract (µg/mL) necessary to prevent oxidative hemolysis of 50% of the erythrocytes population for a ∆t of 60 and 120 min and to prevent lipid peroxidation, respectively. Trolox (Sigma-Aldrich, St. Louis, MO, USA) was the positive control used.

#### 3.4.2. Antimicrobial Activity

The antibacterial activity of the extracts was determined by redissolving them in water to obtain a 100 mg/mL stock solution, being subsequently subjected to successive dilutions. The microdilution method [73] was used against microorganisms from clinical isolates of patients hospitalized in various departments of the Local Health Unit of Bragança and Centro Hospitalar de Trás-os-Montes and Alto-Douro Vila Real, Northeast Portugal. Five Gram-negative bacteria (*Escherichia coli*, *Klebsiella pneumoniae*, *Morganella morganii*, *Proteus mirabilise*, and *Pseudomonas aeruginosa*) and three Gram-positive bacteria (*Enterococcus faecalis, Listeria monocytogenese*, and methicillin-resistant *Staphylococcus aureus* (MRSA)) were used. The results were presented as minimal inhibition concentrations (MICs) and minimal bactericidal concentrations (MBCs). Ampicillin was used as a positive control for all bacterial strains, Imipenemo for all Gram-negative bacteria tested and *L. monocytogenes* and Vancomycin for *Enterococcus faecalis* and MRSA.

#### 3.4.3. Anti-Inflammatory Activity

The extracts were redissolved in water at a concentration of 8 mg/mL and then diluted in the range of 400 to 6.25 µg/mL. A mouse macrophage-like cell line RAW 264.7 was used in this study, the inflammation was induced by LPS, and the Griess Reagent System (GRS) kit was applied to determine the nitric oxide, measured at 540 nm (EL × 800 microplate reader, Bio-Tek Instruments, Inc; Winooski, VT, USA), as described previously [74]. The results were expressed in IC_50_ values (sample concentration providing 50% of inhibition of NO production, μg/mL) and Dexamethasone (50 μM) was used as a positive control, while in negative controls, no LPS was added.

#### 3.4.4. Antitumor Activity

The extracts were re-dissolved in water at a concentration of 8 mg/mL and then diluted in the range of 400 to 6.25 µg/mL. The antitumoral activity was evaluated in five human tumor cell lines: AGS (gastric adenocarcinoma), CaCo-2 (colorectal adenocarcinoma) HeLa (cervical adenocarcinoma), MCF-7 (breast adenocarcinoma), and NCI-H460 (lung carcinoma). The cell lines were plated in 96-well plates, with a final density of 1.0 × 10^4^ cells/mL and were allowed to attach for 24 h. Next, different extract concentrations were added to the cells, which were incubated for 48 h. Both cells treatment and the Sulforhodamine B assay were carried out according to a protocol established by Abreu et al. [75]. All results were expressed as the sample concentration inhibiting 50% of the net cell growth (GI50 values, μg/mL). Ellipticine (Sigma-Aldrich, St. Louis, MO, USA) was applied as the positive control. 

### 3.5. Statistical Analysis

The described tests were performed in triplicate, the results being expressed as mean values ± SD. An analysis of variance (ANOVA) was performed based on the Tukey test with α = 0.05 (when the homoscedasticity of the distributions was verified) or the Tamhane T2 test (heteroscedastic distributions) to classify the statistical differences between the different parameters evaluated. Compliance with ANOVA requirements, specifically the normal distribution of results and the homogeneity of variances, was verified using the Shapiro–Wilk test and the Levene test, respectively. In the remaining cases, the t-Student test was applied, considering a value of α = 0.05 (95% confidence). IBM SPSS Statistics for Windows, version 22.0, was used (IBM Corp., Armonk, NY, USA).

## 4. Conclusions

The results obtained in this study suggest that the studied condiment mixtures are an excellent source of bioactive compounds, supporting the hypothesis that specific phenolic compounds, as well as organic acids and tocopherols, strongly contribute to the bioactive properties of the extracts. Their phenolic composition, especially in apigenin, luteolin, and quercetin derivates confer them high antioxidant and anti-inflammatory capacity and may also be associated with the antitumor and antimicrobial properties against a diversity of microorganisms. Given the wealth of these plant mixtures in valuable bioactive compounds with beneficial effects on human health, their inclusion in daily human diet, as well as their application in food industry, for instance for preserving purposes, is of great importance. Several beneficial effects can be achieved considering the possible cumulative and synergistic effects of all the identified bioactive compounds.

## Figures and Tables

**Table 1:** Nutritional value of the condiment mixtures (g/100 g dw and kcal/100 g dw for energy; mean ± SD).

	Mixture for Poultry	Mixture for Meat	Mixture for Salad	Mixture for Fish
Proteins	18.1 ± 0.2 ^b^	16.82 ± 0.07 ^c^	15.5 ± 0.2 ^d^	20.9 ± 0.3 ^a^
Ashes	9.9 ± 0.1 ^b^	8.9 ± 0.2 ^d^	9.20 ± 0.04 ^c^	10.8 ± 0.3 ^a^
Fat	5.5 ± 0.1 ^a^	3.81 ± 0.03 ^b^	3.294 ± 0.006 ^c^	3.02 ± 0.02 ^d^
Carbohydrates	66.52 ± 0.04 ^c^	70.5 ± 0.2 ^b^	72.0 ± 0.1 ^a^	65.2 ± 0.6 ^d^
Energy	387.534 ± 0.001 ^a^	383.3 ± 0.8 ^b^	379.7 ± 0.2 ^c^	372 ± 1 ^d^

^a, b, c, d^: In each line, different letters represent statistically significant differences (*p* < 0.05).

**Table 2:** Free sugars composition of the condiment mixtures (mg/100 g dw; mean ± SD).

	Mixture for Poultry	Mixture for Meat	Mixture for Salad	Mixture for Fish
Fructose	3.0 ± 0.1 ^b^	0.86 ± 0.03 ^d^	3.4 ± 0.2 ^a^	2.26 ± 0.05 ^c^
Glucose	2.2 ± 0.1 ^b^	1.03 ± 0.03 ^d^	2.7 ± 0.1 ^a^	1.61 ± 0.01 ^c^
Sucrose	1.75 ± 0.04 ^d^	2.38 ± 0.03 ^b^	2.27 ± 0.03 ^c^	2.56 ± 0.04 ^a^
Total	7.0 ± 0.2 ^b^	4.28 ± 0.04 ^d^	8.4 ± 0.3 ^a^	6.427 ± 0.004 ^c^

^a, b, c, d^: In each line, different letters represent statistically significant differences (*p* < 0.05). Equations of the calibration lines obtained with commercial standards: fructose (y = 1.04x, R^2^ = 0.999; LOD = 0.05 mg/mL; LOQ = 0.18 mg/mL); glucose (y = 0.935x, R^2^ = 0.999; LOD = 0.08 mg/mL; LOQ = 0.25 mg/mL); and sucrose (y = 1.17675x, R^2^ = 0.997; LOD = 0.06 mg/mL; LOQ = 0.30 mg/mL).

**Table 3:** Organic acids composition of the condiment mixtures (mg/100 g dw; mean ± SD).

	Mixture for Poultry	Mixture for Meat	Mixture for Salad	Mixture for Fish
Oxalic acid	1.32 ± 0.01 ^a^	0.94 ± 0.02 ^b^	0.78 ± 0.03 ^c^	0.08 ± 0.01 ^d^
Malic acid	1.38 ± 0.04 ^d^	2.34 ± 0.04 ^a^	1.85 ± 0.04 ^b^	1.64 ± 0.08 ^c^
Citric acid	0.95 ± 0.03 ^b^	0.88 ± 0.02 ^b^	nd	1.25 ± 0.07 ^a^
Fumaric acid	tr	tr	nd	nd
Total	3.65 ± 0.09 ^b^	4.17 ± 0.03 ^a^	2.63 ± 0.07 ^c^	3.6 ± 0.1 ^b^

tr: traces; nd: not detected. ^a, b, c, d^: In each line, different letters represent statistically significant differences (*p* < 0.05). Equations of the calibration lines obtained with commercial standards: oxalic acid (y = 1 × 10^7^x + 23,1891; R^2^ = 0.9999; LOD = 12.55 µg/mL; LOQ = 41.82 µg/mL); malic acid (y = 950,041x + 6255.6; R^2^ = 0.9999; LOD = 36 µg/mL; LOQ = 120 µg/mL); citric acid (y = 1 × 10^6^x − 10,277; R^2^ = 0.9997; LOD = 0.11 µg/mL; LOQ = 0.34 µg/mL); and fumaric acid (y = 1 × 10^8^x + 614,399; R^2^ = 0.9986; LOD = 0.08 µg/mL; LOQ = 0.26 µg/mL).

**Table 4:** Composition of tocopherols of the condiment mixtures (mg/100 g dw; mean ± SD).

	Mixture for Poultry	Mixture for Meat	Mixture for Salad	Mixture for Fish
*α*-Tocopherol	4.33 ± 0.01 ^a^	4.16 ± 0.02 ^b^	1.79 ± 0.01 ^c^	1.7 ± 0.1 ^d^
*β*-Tocopherol	1.19 ± 0.03 ^b^	1.75 ± 0.03 ^a^	0.56 ± 0.03 ^d^	1.14 ± 0.01 ^c^
*γ*-Tocopherol	8.17 ± 0.07 ^c^	11.0 ± 0.2 ^b^	13.85 ± 0.07 ^a^	14.1 ± 0.7 ^a^
*δ*-Tocopherol	0.80 ± 0.04 ^a^	0.134 ± 0.005 ^d^	0.446 ± 0.004 ^b^	0.201 ± 0.007 ^c^
Total	14.5 ± 0.1 ^b^	17.0 ± 0.2 ^a^	16.6 ± 0.1 ^a^	17.1 ± 0.8 ^a^

^a, b, c, d^: In each line, different letters represent significant differences (*p* < 0.05). Equations of the calibration lines obtained with commercial standards: α-tocopherol (y = 1.295x; R^2^ = 0.991; LOD: 18.06 ng/mL, LOQ: 60.20 ng/mL); β-tocopherol (y = 0.396x; R^2^ = 0.992; LOD: 25.82 ng/mL, LOQ: 86.07 ng/mL); γ-tocopherol (y = 0.567x; R^2^ = 0.991; LOD: 14.79 ng/mL, LOQ: 49.32 ng/mL); and δ-tocopherol (y = 0.678x; R^2^ = 0.992; LOD: 20.09 ng/mL, LOQ: 66.95 ng/mL).

**Table 5:** Fatty acids composition of the condiment mixtures (relative%; mean ± SD).

	Mixture for Poultry	Mixture for Meat	Mixture for Salad	Mixture for Fish
C6:0	0.1725 ± 0.0007	0.808 ± 0.008	nd	0.75 ± 0.04
C11:0	1.00 ± 0.02	1.221 ± 0.005	1.071 ± 0.014	0.733 ± 0.007
C12:0	0.21 ± 0.01	0.142 ± 0.001	0.70 ± 0.02	0.138 ± 0.001
C13:0	1.22 ± 0.08	1.251 ± 0.007	1.03 ± 0.02	1.17 ± 0.05
C14:0	1.128 ± 0.003	1.01 ± 0.03	1.57 ± 0.02	0.81 ± 0.02
C15:0	0.816 ± 0.006	0.97 ± 0.01	0.297 ± 0.009	0.211 ± 0.004
C16:0	15.7 ± 0.6	18.51 ± 0.03	18.6 ± 0.9	13.8 ± 0.2
C16:1	1.24 ± 0.02	0.99 ± 0.01	0.810 ± 0.006	1.041 ± 0.008
C17:0	1.060 ± 0.007	1.53 ± 0.04	0.762 ± 0.003	0.96 ± 0.06
C18:0	4.1 ± 0.1	4.67 ± 0.06	6.33 ± 0.02	4.0 ± 0.1
C18:1n9c	2.006 ± 0.01	1.66 ± 0.01	2.63 ± 0.07	1.32 ± 0.01
C18:2n6c	21.1 ± 0.3	18.6 ± 0.3	19.8 ± 0.2	21.47 ± 0.04
C18:3n3	42.7 ± 0.2	39.3 ± 0.2	36 ± 1	42.3 ± 0.8
C20:0	1.189 ± 0.002	1.57 ± 0.04	2.01 ± 0.07	3.3 ± 0.1
C20:1	0.163 ± 0.009	0.192 ± 0.007	0.094 ± 0.003	0.081 ± 0.004
C20:2	0.106 ± 0.004	0.086 ± 0.001	0.067 ± 0.001	0.126 ± 0.006
C21:0	0.106 ± 0.007	0.244 ± 0.002	0.182 ± 0.001	0.122 ± 0.003
C20:4n6	1.215 ± 0.003	1.65 ± 0.01	nd	0.50 ± 0.01
C22:0	1.50 ± 0.05	1.818 ± 0.005	3.314 ± 0.005	2.72 ± 0.01
C20:5n3	0.253 ± 0.005	0.53 ± 0.03	nd	0.332 ± 0.004
C22:2	0.62 ± 0.03	0.78 ± 0.04	0.58 ± 0.02	0.86 ± 0.05
C24:0	2.279 ± 0.009	2.109 ± 0.006	3.39 ± 0.04	3.259 ± 0.002
C24:1	0.1805 ± 0.0007	0.363 ± 0.006	0.334 ± 0.006	0.041 ± 0.001
SFA	30.5 ± 0.5 ^a^	35.85 ± 0.09 ^b^	39 ± 1 ^c^	31.9 ± 0.7 ^d^
MUFA	3.59 ± 0.01 ^a^	3.21 ± 0.02 ^c^	3.87 ± 0.06 ^b^	2.478 ± 0.008 ^d^
PUFA	65.8 ± 0.5 ^a^	60.9 ± 0.1 ^b^	57 ± 1 ^d^	65.5 ± 0.7 ^c^

^a, b, c, d^: In each line, different letters represent statistically significant differences (*p* < 0.05). Caproic acid (C6:0); undecylic acid (C11:0); lauric acid (C12:0); tridecanoic acid (C13:0); myristic acid (C14:0); pentadecylic acid (C15:0); palmitic acid (C16:0); palmitoleic acid (C16:1); margaric acid (C17:0); stearic acid (C18:0); oleic acid (C18:1n9c); linoleic acid (C18:2n6c); α-linolenic acid (C18:3n3); arachidic acid (C20:0); cis-11-eicosenoic acid (C20:1); cis-11,14-eicosadienoic acid (C20:2); heneicosylic acid (C21:0); arachidonic acid (C20:4n6); behenic acid (C22:0); cis-5,8,11,14,17-eicosapentaenoic acid (C20:5n3); cis-13,16-docosadienoic acid (C22:2); lignoceric acid (C24:0); nervonic acid (C24:1); SFA: saturated fatty acids; MUFA: monounsaturated fatty acids; PUFA: polyunsaturated fatty acids.

**Table 6:** Retention times (Rt), wavelengths of maximum absorption in the visible region (λ_max_), mass spectral data, and tentative identification of the phenolic compounds present in the condiment mixtures hydroethanolic extracts.

Peak	Rt (min)	λ_max_ (nm)	[M-H]^−^ *m/z*	MS^2^ *m/z*	Attempted Identification	Reference/Method Used for Identification
1	4.95	270	305	219(14),179(30),125(100)	(Epi)gallocatechin	[43]
2	5.91	323	353	191(100),179(34),173(5),165(5)	3-*O*-Caffeoylquinic acid	[35,36]
3	7.86	322	341	179(100),135(23)	Caffeic acid hexoside	[37]
4	8.9	325	593	575(12),503(35),473(100),383(14),353(21)	Apigenin-*C*-dihexoside	[41]
5	9.37	322	179	135(100)	Caffeic acid	Standard compound
6	10.3	280	325	163(100),119(25)	*p*-Coumaric acid hexoside	[37]
7	11.36	284	325	163(100),119(25)	*p*-Coumaric acid hexoside	[37]
8	12.82	326	725	681(42),519(100),357(23),315(67)	Isorhamnetin-*O*-malonyl-dihexoside	DAD/MS
9	13.84	320	1093	917(100),285(13)	Kaempherol-*O*-glycosyl-(*p*-coumaroyl-hexosyl)hexosyl-glucoronide	[42]
10	15.05	343	463	301(100)	Quercetin-3-*O*-glucoside	Standard compound
11	15.96	263/294/336	421	259(54),153(100),108(10)	4-Hydroxy-7-*O*-(3-hydroxy-4-*O*-glucosylbenzoyl)benzyl alcohol	[38]
12	16.87	346	521	359(100),197(35),179(40),161(98)	Rosmarinic acid hexoside	[39]
13	17.69	345	461	285(100)	Luteolin-7-*O*-glucuronide	[37]
14	18.01	344	447	285(100)	Luteolin-7-*O*-glucoside	Standard compound
15	20.64	328	359	197(35),179(39),161(100)	*cis* Rosmarinic acid	Standard compound
16	21.04	330	359	197(35),179(39),161(100)	*trans* Rosmarinic acid	Standard compound
17	22.41	334	717	537(5),519(100),475(13),339(31)	Salvianolic acid B	[39]
18	23.5	337	593	285(100)	Luteolin-7-*O*-rutinoside	DAD/MS
19	24.28	329	537	493(100),359(62),313(15),295(<5),269(<5),197(5),179(5)	Lithospermic acid A	[39]
20	26.03	336	649	605(34),563(41),269(100)	Apigenin-*O*-malonyl-pentosyl-hexoside	DAD/MS
21	26.97	339	649	605(56),563(31),269(100)	Apigenin-*O*-malonyl-pentosyl-hexoside	DAD/MS
22	27.67	340	679	635(100),593(5),299(51)	Diosmetin-*O*-malonyl-hexosyl-pentoside	DAD/MS
23	28.26	323	493	359(100),313(12),295(5),197(10),179(10)	Salvianolic acid A	[39]
24	31.21	287/313	557	513(5),469(37),349(100),163(34)	*p*-Coumaric acid derivative isomer I	[40]
25	31.66	290/310	557	513(5),469(37),349(100),163(34)	*p*-Coumaric acid derivative isomer II	[40]

**Table 7:** Quantification (mg/g of extract) of the phenolic compounds present in the condiment mixtures hydroethanolic extracts (mean ± SD).

Peak	Mixture for Poultry	Mixture for Meat	Mixture for Salad	Mixture for Fish
**1**	0.57 ± 0.01 ^c^	1.11 ± 0.05 ^a^	0.42 ± 0.01 ^d^	0.76 ± 0.03 ^b^
**2**	1.31 ± 0.01 ^c^	1.46 ± 0.06 ^b^	1.05 ± 0.07 ^d^	2.2 ± 0.1 ^a^
**3**	0.21 ± 0.01	tr	tr	0.162 ± 0.002
**4**	0.63 ± 0.03 ^c^	0.752 ± 0.002 ^a^	0.36 ± 0.01 ^d^	0.68 ± 0.01 ^b^
**5**	0.19 ± 0.01	tr	0.185 ± 0.005	nd
**6**	0.46 ± 0.04 ^a^	0.130 ± 0.002 ^c^	0.128 ± 0.002 ^c^	0.21 ± 0.01 ^b^
**7**	0.31 ± 0.01 ^d^	0.40 ± 0.03 ^c^	0.577 ± 0.003 ^b^	0.87 ± 0.02 ^a^
**8**	4.69 ± 0.02 ^d^	4.900 ± 0.001 ^a^	4.75 ± 0.02 ^c^	4.83 ± 0.02 ^b^
**9**	4.65 ± 0.01 ^b^	4.65 ± 0.01 ^b^	4.66 ± 0.02 ^b^	6.59 ± 0.03 ^a^
**10**	nd	7.8 ± 0.2 ^a^	6.46 ± 0.08 ^b^	5.475 ± 0.001 ^c^
**11**	4.69 ± 0.01^a^	4.69 ± 0.03 ^a^	4.66 ± 0.01 ^b^	4.69 ± 0.01 ^a^
**12**	0.67 ± 0.01 ^b^	2.9 ± 0.2 ^a^	0.384 ± 0.003 ^c^	2.94 ± 0.02 ^a^
**13**	4.92 ± 0.06 ^c^	5.38 ± 0.04 ^a^	5.3 ± 0.1 ^b^	5.39 ± 0.08 ^a^
**14**	4.9 ± 0.1 ^c^	7.35 ± 0.07 ^a^	4.98 ± 0.02 ^c^	5.62 ± 0.04 ^b^
**15**	6.0 ± 0.2 ^b^	10.7 ± 0.6 ^a^	1.47 ± 0.06 ^d^	2.17 ± 0.05 ^c^
**16**	6.8 ± 0.4 ^b^	14.6 ± 0.5 ^a^	2.53 ± 0.07 ^d^	4.2 ± 0.1 ^c^
**17**	0.54 ± 0.02 ^d^	0.58 ± 0.03 ^c^	0.63 ± 0.01 ^b^	1.36 ± 0.04 ^a^
**18**	4.7 ± 0.1 ^b^	4.79 ± 0.05 ^a^	4.67 ± 0.01 ^c^	4.75 ± 0.01 ^b^
**19**	0.49 ± 0.03 ^c^	0.85 ± 0.02 ^a^	0.49 ± 0.02 ^c^	0.61 ± 0.03 ^b^
**20**	17.3 ± 0.9 ^b^	23.5 ± 0.9 ^a^	6.3 ± 0.3 ^c^	17.6 ± 0.3 ^b^
**21**	0.89 ± 0.02 ^b^	1.4 ± 0.1 ^a^	0.64 ± 0.02 ^d^	0.85 ± 0.04 ^c^
**22**	1.12 ± 0.04 ^b^	1.22 ± 0.02 ^a^	0.64 ± 0.05 ^d^	1.064 ± 0.002 ^c^
**23**	0.99 ± 0.07 ^b^	0.76 ± 0.06 ^c^	0.43 ± 0.01 ^d^	2.1 ± 0.1 ^a^
**24**	0.427 ± 0.002 ^b^	0.53 ± 0.03 ^a^	0.16 ± 0.01 ^d^	0.43 ± 0.02 ^c^
**25**	0.516 ± 0.004 ^c^	0.738 ± 0.003 ^a^	0.25 ± 0.01 ^d^	0.56 ± 0.02 ^b^
Total Phenolic Acids	23.6 ± 0.7 ^b^	39.4 ± 0.4 ^a^	12.9 ± 0.2 ^d^	22.46 ± 0.04 ^c^
Total Flavonoids	45 ± 1 ^c^	61.8 ± 0.9 ^a^	39.1 ± 0.6 ^d^	53.6 ± 0.6 ^b^
Total	68 ± 2 ^c^	101.2 ± 0.6 ^a^	52.1 ± 0.8 ^d^	76.1 ± 0.6 ^b^

tr: traces; nd: not detected; Calibration curves used: chlorogenic acid (y = 168823x − 161172; R^2^ = 0.9999, LOD (Limit of detection) = 0.20 µg/mL and LOQ (Limit of quantification) = 0.68 µg/mL peak 2); *p*-coumaric acid (y = 301,950x + 6966.7, R^2^ = 0.9999, LOD = 0.68 μg/mL; LOQ = 1.61 μg/mL, peaks 6, 7, 24 and 25); rosmarinic acid (y = 191291x − 652,903, R^2^ = 0.999, LOD = 0.15 µg/mL; LOQ = 0.68 µg/mL peaks 12, 15, 16, 17, 19 and 23); caffeic acid (y = 388,345x + 406,369, R^2^ = 0.99; LOD = 0.78 µg/mL; LOQ = 1.97 µg/mL, peaks 3 and 5); apigenin-6-*C*-glucoside (y = 107,025x + 61,531, R^2^ = 0.998; LOD = 0.19 µg/mL; LOQ = 0.63 µg/mL peak 4); apigenin-7-*O*-glucoside (y = 10,683x − 45,794, R^2^ = 0.996, LOD = 0.10 µg/mL; LOQ = 0.53 µg/mL, peaks 20, 21 and 22); (+)-catechin (y = 84,950x − 23,200, R^2^ = 1, LOD = 0.17 μg/mL; LOQ = 0.68 μg/mL, peak 1); quercetin-3-*O*-glucoside (y = 34,843x − 16,0173, R^2^ = 0.9999, LOD = 0.21 μg/mL; LOQ = 0.71 μg/mL, peaks 8, 9, 10, 11, 13, 14 and 18). ^a, b, c, d^: In each line, different letters represent statistically significant differences (*p* < 0.05). *p*-value resulting from the Student *t* test: < 0.001 (peak 3); < 0.001 (peak 5).

**Table 8:** Antioxidant activities of the condiment mixtures hydroethanolic extracts (IC50, µg/mL; mean ± SD).

	Mixture for Poultry	Mixture for Meat	Mixture for Salad	Mixture for Fish
TBARS	27 ± 2 ^c^	4.8 ± 0.3 ^a^	6.6 ± 0.4 ^a^	23 ± 2 ^b^
OxHLIA 60 min	16.5 ± 0.8 ^a^	13 ± 2 ^a^	68 ± 2 ^b^	106 ± 8 ^c^
OxHLIA 120 min	na	na	98 ± 6	na

na: no activity. ^a, b, c, d^: In each line, different letters represent significant differences (*p* < 0.05). Trolox IC_50_ values (positive control): 5.4 ± 0.3 µg/mL (TBARS); 21.8 ± 0.3 µg/mL (OxHLIA 60 min); 43.5 ± 0.3 µg/mL (OxHLIA 120 min).

**Table 9:** Antimicrobial activity (minimal inhibition concentration (MIC) and minimal bactericidal concentration (MBC); mg/mL) of the condiment mixtures hydroethanolic extracts.

	Mixture for Poultry	Mixture for Meat	Mixture for Salad	Mixture for Fish	Ampicillin *		Imipenem *		Vancomicin *	
	MIC	MIC	MIC	MIC	MIC	MBC	MIC	MBC	MIC	MBC
*E. coli*	5	10	5	5	< 0.15	< 0.15	< 0.0078	< 0.0078	nt	nt
*K. pneumoniae*	> 20	>20	> 20	> 20	10	20	< 0.0078	< 0.0078	nt	nt
*M. morganii*	5	2.5	5	10	20	> 20	< 0.0078	< 0.0078	nt	nt
*P. mirabilis*	10	10	10	20	< 015	< 0.15	< 0.0078	< 0.0078	nt	nt
*P. aeruginosa*	> 20	>20	> 20	> 20	> 20	> 20	0.5	1	nt	nt
*E. faecalis*	5	2.5	2.5	5	< 0.15	< 0.15	nt	nt	< 0.0078	< 0.0078
*L. monocytogenes*	20	10	10	10	< 0.15	< 0.15	< 0.0078	< 0.0078	nt	nt
MRSA	5	2.5	5	10	< 0.15	< 0.15	nt	nt	0.25	0.5

Minimum inhibitory concentration (MIC); minimum bactericidal concentration (MBC); methicillin resistant Staphylococcus aureus (MRSA); nt-not tested. * positive control.

**Table 10:** Anti-inflammatory and antitumor activity (IC_50_ values μg/mL) of the condiment mixtures hydroethanolic extracts (mean ± SD).

	Mixture for Poultry	Mixture for Meat	Mixture for Salad	Mixture for Fish
**Anti-inflammatory activity**				
RAW 264.7	54 ± 2 ^a^	149 ± 5 ^b^	> 400	59 ± 3 ^a^
**Antitumor activity**				
AGS	261 ± 5 ^c^	184 ± 12 ^b^	98 ± 2 ^a^	301 ± 4 ^d^
HeLa	292 ± 3 ^b^	327 ± 22 ^c^	88 ± 1 ^a^	294 ± 2 ^b^
MCF-7	183.3 ± 0.2 ^c^	53.25 ± 0.02 ^b^	10.54 ± 0.02 ^a^	215 ± 5 ^d^
NCI-H460	341.7 ± 0.2 ^b^	> 400	318 ± 1 ^a^	348 ± 2 ^c^
CaCo-2	238.9 ± 0.5 ^b^	238 ± 2 ^b^	192 ± 1 ^a^	294.01 ± 0.03 ^c^

^a, b, c, d^: In each line, different letters represent significant differences (*p* < 0.05). Dexamethasone GI_50_ value (positive control): 6.3 ± 0.4 µg/mL. Elipticin GI_50_ values (positive control): 0.22 ± 0.02 µg/mL (AGS); 0.25 ± 0.02 µg/mL (HeLa); 0.251 ± 0.001 µg/mL (MCF-7); 0.249 ± 0.002 µg/mL (NCI-H460); 0.20 ± 0.02 µg/mL (CaCo-2).

## Data Availability

The data presented in this study are available in this article.

## Sample Availability

Samples of the condiment mixtures are available from the authors.
